# Round the Hospitals

**Published:** 1921-09-03

**Authors:** 


### ROUND THE HOSPITALS.

There is still time for nurses, who by reason of disablement due to war service are unable to continue nursing, to obtain free training for some other occupation under the scheme formulated by the Ministry of Labour. The scheme allows for the payment of training fees and maintenance allowance, in addition to the pension to which the disabled nurse may be entitled. Applications must be made to the Controller, Women's Training Branch, Ministry of Labour, St. Ermin's, Caxton Street, S.W. 1, before October 31 next, and the only extension of time that will be allowed is in the case of (*a*) nurses demobilised after that date, and (*b*) nurses who on that date are in attendance either at a convalescent centre of the Pensions Ministry or are in-patients at a Military or Pensions hospital. In the former case (*a*) such nurses may apply within three months of the date of demobilisation, and in the latter case (*b*) within three months of discharge from the centre or hospital.

Representatives of the local authorities in Scotland held a meeting recently to urge the amendment of the rules framed by the General Nursing Council for Scotland under the Nurses' Registration Act. The contention is that nurses trained in hospitals maintained by local authorities under the Public Health Act, fever hospitals, in short, should be admitted to the general part of the Register, and that the Supplementary Register of fever nurses be abolished. The Secretary of State for Scotland is to be asked to receive a deputation on the subject.

The Convener of Aberdeen County, Mr. H. D. McCombie, is warmly promoting a scheme for completing the county nursing service. There are already twenty-six district nursing associations in various parishes in Aberdeenshire, under the Jubilee Institute, ready to join in the scheme, and it is proposed to divide the county into fifty areas, with convenient centres. The main difficulty relates to the scarcity of nurses. Three of the existing associations are suspended for lack of a nurse, and seven more nurses are urgently needed at once, which the Jubilee Institute at Edinburgh is unable to supply. Salaries have increased and working hours have been reduced, so that the outlook for the future is more hopeful. But we believe salaries will have to go still higher before the needs of the country districts can be adequately supplied.

We have received the annual report of the General Hospital, Nottingham, and note that the board, having received a communication from the College of Nursing recommending a minimum scale of salaries for members of the nursing staff, have adopted this in a modified form, and have sanctioned an additional annual expenditure on nurses' salaries of £725 for the first year, with a further addition of £95 for the second year. Plans have been approved for the new nurses' home, and the work is to be commenced next year if possible. We regret to find no report from the Matron on the year's work in the training-school and other matters affecting the nursing staff.

The Conference which the Chartered Society of Massage and Medical Gymnastics proposes to hold on October 6, 7, and 8 will touch on very interesting subjects, and should command a good attendance. Sir James Purves Stewart will lecture on "Disseminated Sclerosis"; Dr. Hurst on "Massage and Exercises in Gastro-Intestinal Disorders"; Sir Henry Gauvain on "Surgical Tuberculosis, and Indications for Massage"; Dr. H. Crichton Miller on "The Psychological Significance of Rest"; and Miss Stansfeld on "The Importance of Physical Fitness for the Professional Woman." The sittings will be at 93 Mortimer Street, Steinway Hall, and Guy's Hospital respectively. Books of tickets, transferable to other members, for the whole Conference can be obtained by members of the Society at 7*s.* 6*d.* from the Secretary, 157 Great Portland Street, W. 1.

Bermondsey Board of Guardians have decided to extend the training period for the probationer nurses at their infirmary to four years. Dr. Harkness, the medical superintendent, has pointed out that four years is in force in many general hospitals and in a few Poor-Law infirmaries, and difficulty is at present experienced in the completion of the curriculum in the time available. He feels that in three years it will be impossible to pay more than lip service to the new syllabus issued by the General Nursing Council, particularly in view of the fact that many of the probationers, by reason of defective primary education, do not make very rapid progress at first. All future appointments of probationer nurses will be for four years, whilst those at present will have the option of extending their three years' agreements to four years.

The introductory lecture to the students taking one of the Royal Sanitary Institute courses in the coming session will be delivered at 90 Buckingham Palace Road, S.W. 1, by Professor H. R. Kenwood on Monday, September 19, at 5.30, and admission will be free. The ensuing course for sanitary officers commences on September 21, that for meat and food inspectors on September 30, and that for women health visitors and child welfare workers on September 23. Candidates for the latter course must produce evidence of practical training and experience, including nursing, as required by the regulations for each examination. This course is well fitted to be of service to nurses on passing out of their training schools to take up Public Health work. Its value is enhanced by the demonstrations, which include attendance at six infant consultations under Dr. Eric Pritchard and visits to various places of interest.

Male nurses are not greatly enamoured of their title, and one openly protests in a contemporary against the use of the word "nurse" in the masculine gender. We cordially agree that the title is one which should be sacred to women, but it is difficult to find a satisfactory equivalent. Of the two terms suggested for men, "sick attendant" and "nursing orderly," we greatly prefer the latter. The need is for a name which distinctly implies a term of training and the attainment of a definite standard.

Women in South Australia are using the influence they gained during the recent elections to promote the welfare of women workers generally. In particular they have been examining into the conditions of labour imposed on nurses, and are urging reform in the administration of both public and private hospitals. They press for the specification of maximum hours on duty, for better housing accommodation for nurses, and for limiting the number of probationers employed in fixed proportion to the number of qualified nurses. They are also urging on the Government the desirability of licensing, classifying, and inspecting all private hospitals or nursing homes. We anticipate much from the interest in nursing matters which has been roused in liberal circles.

